# Health Service Utilization and Poor Health Reporting in Asthma Patients

**DOI:** 10.3390/ijerph13070645

**Published:** 2016-06-30

**Authors:** Joshua G. Behr, Rafael Diaz, Muge Akpinar-Elci

**Affiliations:** 1 Virginia Modeling, Analysis and Simulation Center, Old Dominion University, Norfolk, VA 23529, USA; 2 MIT-Zaragoza Logistics Center, Massachusetts Institute of Technology, Zaragoza 50197, España; diazr@mit.edu; 3 Center for Global Health, Old Dominion University, Norfolk, VA 23529, USA; makpinar@odu.edu

**Keywords:** asthma, utilization, frequent utilization, emergency department, primary care, hospital, poor health, chronic condition

## Abstract

The management and treatment of adult asthma has been associated with utilization of health services. *Objectives*: First, to investigate the likelihood of health service utilization, including primary care, emergency department, and hospital stays, among persons diagnosed with an asthma condition relative to those that do not have an asthma condition. Second, to examine the likelihood of poor physical health among asthma respondents relative to those that do not have an asthma condition. Third, to demonstrate that these relationships vary with frequency of utilization. Fourth, to discuss the magnitude of differences in frequent utilization between asthma and non-asthma respondents. *Data Source*: Data is derived from a random, stratified sampling of Hampton Roads adults, 18 years and older (*n* = 1678). *Study Design*: Study participants are interviewed to identify asthma diagnosis, access to primary care, frequency of emergency department utilization, hospital admissions, and days of poor physical health. Odds-ratios establish relationships with the covariates on the outcome variable. *Findings*: Those with asthma are found more likely (OR 1.50, 95% CI 1.05–2.15) to report poor physical health relative to non-asthma study participants. Further, asthma respondents are found more likely (OR 4.23, 95% CI 1.56–11.69) to frequently utilize primary care that may be associated with the management of the condition and are also more likely to utilize treatment services, such as the emergency department (OR 1.87, 95% CI 1.32–2.65) and hospitalization (OR 2.21, 95% CI 1.39–3.50), associated with acute and episodic care. Further, it is a novel finding that these likelihoods increase with frequency of utilization for emergency department visits and hospital stays. *Conclusion*: Continuity in care and better management of the diseases may result in less demand for emergency department services and hospitalization. Health care systems need to recognize that asthma patients are increasingly more likely to be characterized as frequent utilizers of both primary and emergency department care as the threshold for what constitutes frequent utilization increases. Investments in prevention and better management of the chronic condition may result in less demand for acute care services, especially among high frequency utilizers.

## 1. Introduction

Asthma is a chronic inflammatory disease that broadly encompasses airway obstruction, hyper-responsiveness, and inflammation [1]. Asthma may be triggered by any number of environmental conditions, airborne particulate matter, and occupational conditions [2]. It is estimated that nearly 235 million people world-wide suffer from asthma [3] and globally there is evidence that the prevalence of asthma may be increasing [4,5]. Within the U.S., the prevalence of asthma among adults is nearly 9% and the prevalence of asthma at some time in the lifespan (lifetime asthma) is nearly 14%, meaning asthma has impacted over 34 million adult Americans [6].

The treatment and management of asthma, as well as asthma-associated episodic events, necessitates access to health professionals and utilization of health system resources [7,8]. Routine office visits for asthma, asthma-related hospital stays, emergency department visits, and urgent care visits involved in asthma control and management constitute health service utilization. Uncontrolled asthma is associated with more urgent health care utilization and failure to control, especially among those with severe asthma, contributes to frequent utilization of emergency services [9,10].

While there are different levels of asthmatic severity, many who suffer from mild intermittent and mild persistent asthma associated with inflammation may control the symptoms through therapy [11,12,13]. The failure to properly coordinate asthma-related drug therapy regimens has been associated with increased healthcare utilization, including emergency department visits [14,15,16,17,18,19,20,21,22] and hospitalization [23,24]. Failure to adhere to asthma treatment regimens has been shown to escalate healthcare costs [25,26,27,28].

In addition, asthmatics with better asthma control report better overall physical health relative to those with poor asthma control [29,30,31,32,33,34,35,36]. For example, adults that are obese are more likely to report asthma symptoms relative to non-obese counterparts [35,37,38,39,40]. In addition, those with poor overall physical health are more likely to experience asthma exacerbation and subsequent emergency department utilization [41,42,43].

Government statistics report the prevalence of adult asthma to be 8.7% across Virginia and 8.9% within the urban south side portion of Hampton Roads [44]. We expect, through our sampling design, to identify similar asthma prevalence in Hampton Roads. We also expect, similar to existing research, that asthma will be associated with higher health care utilization and poor physical health. In this present study, we investigate the connection between asthma and frequent health service utilization. Since there is not a consensus on what constitutes frequent utilization [45], we examine likelihoods across a range of utilization levels (*aka* utilization thresholds). 

The objectives of this study are several fold. Frist, to investigate the likelihood of health service utilization, including primary care, emergency department, and hospital stays, among persons diagnosed with an asthma condition relative to those that do not have an asthma condition. Second, to examine the likelihood of poor physical health among asthma respondents relative to those that do not have an asthma condition. Third, to demonstrate that these relationships vary with frequency of utilization. Fourth, to discuss the magnitude of differences in frequent utilization between asthma and non-asthma respondents. 

## 2. Population and Design

The data for this study are derived from a random sampling of 1678 adults (18+ years of age) stratified across 16 localities (17.26% response rate) in the Hampton Roads region in Southeast Virginia, an area encompassing nearly 1.9 million citizens. Trained research associates administered a questionnaire in which study participants were asked to self-report primary care visits, emergency department visits, admittance to a hospital, overall health, and asthma diagnosis. The representativeness of the sampling is validated, in part, by noting that within the sample 8.8% are recorded as having the condition of asthma. The CDC estimates adult asthma within the State to be 8.7% (CI 7.9%–9.5%), which is very similar to our findings [1]. The study design was vetted and approved by the Institutional Review Board (ODURF-562371). 

### 2.1. Data Management and Analysis

Relationships with the covariates (predictor variables) on the outcome variable, asthma, are tested using logistic regression to establish odds-ratios. The outcome variable is measured as a dichotomous response to the question, “*Could you please tell me, yes or no, whether a doctor or nurse within the past 5 years has told you that you have asthma?*” This question wording is intended to elicit from respondents the diagnosis of asthma from a health care professional, although it is noted that such self-recollection may not be perfect. 

Differences in health service utilization is measured by asking study participants to state frequency of encounters with the health system. To wit, “*How many times in the past year have you gone to the emergency room for yourself?*”, “*How many times in the past year, not counting emergency room visits, were you seen by a doctor or nurse to get care?*”, and “*How many times within the past year did you spend the night in the hospital other than for childbirth?*” For each of these questions, five covariates are created. For each of these covariates the control (baseline) category is zero representing the absence of an encounter with the health system within the past year. The intervention, coded as one, is the occurrence of either one or more, two or more, three or more, four or more, or five or more encounters with the health system. Formulating these utilization variables as dichotomous (rather than continuous) as well as coding these variables as “…or more” rather than “…or less” is appropriate. The intent of the research is not only to explore the relative likelihood of utilization among asthma respondents, but also to illustrate differences among frequent service utilizers; frequency of utilization may be conceptualized as a threshold above which subjects encounter the service. Utility is found in not exploring relationships at discrete points (i.e., 1, 2, 3, 4, or 5 visits) but, rather, in exploring thresholds defined as frequency of visits beyond a discrete point. In this context, coding the frequency utilization variables as one or more, two or more, three or more, four or more, and five or more is justified. Also, it is notable that for any single utilization variable (primary care visits, emergency department visits, and hospital stays) the cases falling within these five covariates may be different. Therefore, caution should be used in making comparative statement among the various odds-ratios, even when appearing in the same table.

Difference in physical health is measured by eliciting from the respondent an estimate for the number of recent days when physical health was poor: “*Now thinking about your physical health, which includes physical illness and injury, for how many days during the past 30 days was your physical health not good?”* Similar to the methodology above, the control category is zero, representing the absence of any days where physical health is not good, and the five covariates each representing interventions where physical health is recorded as not good one or more, two or more, three or more, four or more, and five or more days. This physical health variable is conceptualized and measured in a fashion similar to the above utilization variables.

### 2.2. Sample Characteristics

Table 1 presents statistics for the sample for the descriptive variables gender, age, and race/ethnicity, controlled for by diagnosed asthma/no reported asthma.

## 3. Results

Table 2 reports the associations among asthma and utilization for several healthcare services, including primary care, emergency department, and hospital stays.

Study participants with asthma are 323% more likely (OR 4.23, 95% CI 1.56–11.69) to report one or more visits to a primary care doctor relative to those respondents that do not have asthma. As the frequency of utilization increases, the likelihood that study participants with asthma will utilize primary care more frequently than study participants without asthma also increases. The odds-ratios increase from 4.23 (one or more visits) to 5.93 (five of more visits). Substantively, this suggests that those with asthma are more likely to use primary care, especially when there is a pattern of high frequency utilization.

Respondents with asthma are also more likely to utilize the emergency department more frequently relative to those without asthma. Table 2 reports that those with asthma are 87% more likely (OR 1.87, 95% CI 1.32–2.65) relative to those without asthma to have at least one or more emergency department visits within the past year and this propensity is pronounced with increased utilization. While asthma respondents are roughly 87% more likely relative to non-asthma respondents to visit the emergency department one or more times, the odds of an asthma respondent being categorized as a frequent utilizer of emergency services is, for example, over fivefold that of non-asthma respondents when defined as five or more visits.

In similar fashion, respondents with asthma report more frequent stays within the hospital relative to those without asthma. Asthma respondents are roughly 2–3 times as likely to have more frequent hospital stays (1 through 5 or more days) relative to non-asthma respondents.

Study participant were also queried about the status of their general physical health by reporting the number of days within the past 30 days that health was not good. The expectation is that those diagnosed with asthma may physically suffer discomfort stemming from the condition and have the perception that their physical health was not good for one or more days within the past month. Table 3 reports the likelihood that asthma respondents will report poor physical health relative to non-asthma respondents. Asthma respondents are 78% more likely (OR 1.78, 95% CI 1.18–2.68) than non-asthma respondents to report health not good for five or more days. The likelihoods decrease somewhat as we approach two or more days. However, there is statistically little difference between asthma and non-asthma respondents who report health being not good for one or more days. 

## 4. Discussion

The treatment and management of asthma-related conditions requires the utilization of both primary care and episodic care health services [7]. While this is intuitive, of note here is not only the extent of this health service utilization by asthmatic persons relative to non-asthmatic persons, but also the change in likelihood as frequency increases. While such comparisons must be qualified because the cases within each analysis vary somewhat, these changes are modest and do not alter the general point being advanced. As illustrated in Table 2 above, asthma respondents are three-to-four times more likely to utilize primary care relative to non-asthma respondents, but the range of odds-ratios (4.23–5.93) increases by 270% (i.e., likelihood increases by a magnitude of 2.7 across this range). This suggests that asthma respondents not only are more likely to seek services from primary care but, in addition, are also much more likely to be *frequent* utilizers of primary care. In other words, asthma respondents, relative to non-asthma respondents, are increasingly more likely to be characterized as frequent utilizers as the threshold for what constitutes frequent utilization increases. We note again, though, that a specific definition of what number of healthcare visits may constitute “frequent” utilization lacks consensus [46,47,48,49]. The utility of providing a range of thresholds is demonstrated here as one may see changes in odds as utilization increases.

The emergency department generally serves a stable population with relatively few emergency department presentments from new patients [50]. Within the U.S., there are nearly 43 emergency department visits per 100 residents, cumulating to 130 million visits annually, suggesting that emergency department presentations among the general population are not unusual [51]. Stemming from the understanding that those diagnosed with asthma are more likely to have an episodic event relative to the general population, our expectation (and our finding) is that there are differences in frequency of emergency department utilization between asthma and non-asthma respondents [52,53]. A contribution is noted, again, in that the odds-ratio range from 1.87 to 6.35, increasing by a magnitude of 4.5 across this range. This suggests that asthma respondents, relative to non-asthma respondents, are much more likely to be characterized as frequent utilizers of the emergency department as the utilization threshold increases. 

With increased odds of frequent emergency department utilization for asthma respondents, we may expect concomitant admittance to the hospital. While hospital stays are more likely for asthma respondents relative to non-asthma respondents, it is notable that similar magnitudes for the odds-ratios are not necessarily found for hospital stays, which range from 2.21 to 3.53. In other words, while asthma respondents, relative to non-asthma respondents, are much more likely to seek primary care and visit the emergency department, they are relatively more similar to non-asthma respondents when it comes to hospital stays. 

Self-reported fair or poor health has been associated with increased utilization of health services [54,55,56]. Since the condition of asthma, even in its mild intermittent form, may interfere with activities of daily living, it is reasonable to expect asthma respondents to be more likely to report at least a single day where physical health has not been good. Our results show that, when aggregated within a simple dichotomous measure of one or more visits, asthma respondents are not any more likely than non-asthma respondents to report one or more days of poor physical health. However, this aggregation may mask differences; notable is the increase in odds-ratio beyond two or more visits. This suggests that asthma respondents are more likely than non-asthma respondents to report frequent days of poor physical health, especially as the number of days increase.

## 5. Conclusions

We have demonstrated that study participants that have been diagnosed with an asthma-related condition are both more likely to report poor health (in terms of days where health is not good) and more likely to utilize health services relative to those that do not report being diagnosed with asthma. In particular, we have demonstrated that asthmatic participants, relative to non-asthmatic participants, are not only more likely to frequently utilize primary care that may be associated with the management of the condition, but also are more likely to utilize treatment services associated with acute and episodic care, such as emergency department and hospital stays.

In addition, this research has made a novel contribution by refining our understanding that asthma patients are increasingly more likely to be characterized as frequent utilizers of both primary and emergency department care as the threshold for what constitutes frequent utilization increases. It is also a novel finding that the magnitude of the likelihoods are more modest for hospital stays relative to emergency department visits. The implication is that continuity in care, appropriate prevention programs, and better management of the diseases, especially for those that demonstrate high frequent utilization, may result in less demand for acute care services.

## Figures and Tables

**Table 1:** Descriptive characteristics of the sample.

Descriptive Varriable & Attibutes	Combined		Diagnosed Asthma		No Reported Asthma	
	No. (%)		No. (%)		No. (%)	
**Gender**						
Male	532	(31.7)	27	(18.2)	505	(33.0)
Female	1146	(68.3)	121	(81.8)	1025	(67.0)
**Age (year)**						
29 years or less	260	(15.5)	32	(21.6)	228	(14.9)
30 to 39 years	296	(17.6)	22	(14.9)	274	(17.9)
40 to 49 years	378	(22.5)	26	(17.6)	352	(23.0)
50 to 59 years	335	(20.0)	28	(18.9)	307	(20.1)
60 or more years	392	(23.4)	38	(25.7)	354	(23.1)
Refuse	17	(1.0)	2	(1.4)	15	(1.0)
**Race/Ethnicity**						
White/Anglo/Caucasian	1190	(70.9)	89	(60.1)	1101	(72.0)
Black/African American	383	(22.8)	50	(33.8)	333	(21.8)
Hispanic/Latin American	31	(1.9)	4	(2.7)	27	(1.8)
Asian/Pacific Islander	24	(1.4)	2	(1.4)	22	(1.4)
Other	39	(2.4)	3	(2.0)	36	(2.4)
Refuse	10	(0.7)	0	(0.0)	10	(0.7)

**Table 2:** Utilization of healthcare services by asthma respondents.

Utilization Type & Frequency	Odds Ratio	95% CI	C&S ^1^, Nag. ^2^	*p* Sig. ^3^
**Primary Care**				
1 or more visits	4.23	1.56–11.69	0.008, 0.017	0.005
2 or more visits	4.71	1.72–12.90	0.011, 0.023	0.003
3 or more visits	5.41	1.97–14.88	0.016, 0.034	0.001
4 or more visits	5.79	2.09–16.01	0.022, 0.045	0.001
5 or more visits	5.93	2.12–16.54	0.028, 0.058	0.001
**Emergency Department**				
1 or more visits	1.87	1.32–2.65	0.007, 0.016	0.000
2 or more visits	3.57	2.35–5.40	0.022, 0.049	0.000
3 or more visits	3.47	1.97–6.09	0.012, 0.027	0.000
4 or more visits	5.33	2.70–10.54	0.014, 0.033	0.000
5 or more visits	6.35	2.99–13.52	0.014, 0.034	0.000
**Hospital Stays**				
1 or more days	2.21	1.39–3.50	0.009, 0.018	0.001
2 or more days	3.10	1.85–5.19	0.015, 0.030	0.000
3 or more days	3.53	2.00–6.22	0.015, 0.031	0.000
4 or more days	2.33	1.09–4.97	0.004, 0.008	0.029
5 or more days	2.81	1.25–6.35	0.005, 0.011	0.013

**^1^** Cox & Snell; **^2^** Nagelkerke; **^3^** Significance.

**Table 3:** General physical health by asthma respondents.

Physical Health & Frequency	Odds Ratio	95% CI	C&S ^1^, Nag. ^2^	*p* Sig. ^3^
**Health Not Good**				
1 or more days	1.37	0.97–1.95	0.002, 0.004	0.078 ^†^
2 or more days	1.50	1.05–2.15	0.003, 0.007	0.026
3 or more days	1.64	1.21–2.39	0.004, 0.009	0.011
4 or more days	1.66	1.11–2.48	0.004, 0.009	0.014
5 or more days	1.78	1.18–2.68	0.005, 0.011	0.006

**^1^** Cox & Snell; **^2^** Nagelkerke; **^3^** Significance; ^†^ Statistically no significant difference between asthma and non-asthma respondents.
